# Predictors of psychological distress in Syrian refugees with posttraumatic stress in Germany

**DOI:** 10.1371/journal.pone.0254406

**Published:** 2021-08-04

**Authors:** Anna Renner, David Jäckle, Michaela Nagl, Rahel Hoffmann, Susanne Röhr, Franziska Jung, Thomas Grochtdreis, Judith Dams, Hans-Helmut König, Steffi Riedel-Heller, Anette Kersting

**Affiliations:** 1 Department of Psychosomatic Medicine and Psychotherapy, University of Leipzig, Leipzig, Germany; 2 Institute of Social Medicine, Occupational Health and Public Health (ISAP), Medical Faculty, University of Leipzig, Leipzig, Germany; 3 Department of Health Economics and Health Services Research, University Medical Center Hamburg-Eppendorf, Hamburg, Germany; University of Vienna, AUSTRIA

## Abstract

Syria has been the main country of citizenship of refugees in Germany since 2013. Syrians face numerous human rights violations in their country that can be accompanied by the experience of potentially traumatic events, loss and displacement. Along the migration process, refugees are exposed to various factors that can have an impact on mental health. The aim of this study is to investigate sociodemographic, war- and flight-related as well as post-migration factors as predictors of posttraumatic stress, depression, somatization and anxiety in Syrian refugees with posttraumatic stress symptoms based in Germany. Data were based on the baseline sample of the “Sanadak” randomized-controlled trial. A total of 133 adult Syrian refugees participated in the study. A questionnaire covered sociodemographic and flight-related questions as well as standardized instruments for symptoms of PTSD (PDS-5), depression (PHQ-9), somatization (PHQ-15), anxiety (GAD-7), generalized self-efficacy (GSE), religiousness (Z-Scale), social support (ESSI) and mental health stigma (SSMIS-SF). Linear regression models were executed to predict mental health outcomes. Sociodemographic predictors (i.e., female sex, higher education) and flight-related predicting factors (i.e., variability of traumatic events) have a negative impact on mental health in Syrian refugees with posttraumatic stress symptoms in Germany. Mental health stigma predicts worse mental health outcomes. Post-migration factors have a major impact on mental health, such as low income, lack of social support, low life satisfaction or a strongly felt connection to Syria. Somatization is an important manifestation of mental distress in Syrian refugees with posttraumatic stress symptoms. Our study showed a range of factors predicting the mental health of Syrian refugees with posttraumatic stress symptoms. Measures to foster mental health could be securing financial security, promoting gender equality and tailored psychosocial programs addressing mental health stigma, loss and social support networks.

## Introduction

The Syrian war is one of the biggest humanitarian crises of our time, with over 5.5 million people from Syria being registered as refugees worldwide [1, 2]. Main countries of arrival of Syrian refugees remain Turkey, Lebanon and Jordan [2]. Despite the comparably small numbers of asylum seekers from Syria in European countries, Syria has been the main country of citizenship of refugees within Europe and Germany since 2013, representing 12.1% of all refugees in Europe in 2019 [3]. Germany is the main country of destination in Europe, with 36.433 asylum applications from Syria in 2020, which accounts for 35.5% of all applications in this year [4].

Syrian people face numerous human rights violations in their country that can be accompanied by the experience of potentially traumatic events (TE), loss and displacement [5, 6]. Examples of potentially traumatic events that Syrian refugees may have had to endure include hostage-taking, torture, murder, massacres and sexual violence [7]. In addition, throughout the migration process as well as in the post-migration phase refugees are exposed to various factors (e.g., limited access to food, sanitation, housing, health care, education) that can have an impact on mental health [7].

### Mental distress in Syrian refugees

Prevalence rates of mental distress in Syrian refugees are shown to be high, but differ widely between studies due to a variety of factors such as study country, time of assessment or study methodology [8, 9]. A population-based survey from Sweden, which might be comparable to Germany regarding post-migration factors in European high-income-countries, reported prevalence rates of 29.9% for PTSD, 40.2% for depression, 31.8% for anxiety and 37.7% for low subjective well-being in Syrian refugees, together with high numbers of comorbidities [10]. Lower rates were found in a registry-based study in Germany by Georgiadou et al. [11] with 11.4% for PTSD, 14.5% for moderate to severe depression and 13.5% for moderate to severe generalized anxiety. In comparison to the other outcomes, only a few representative studies assessed somatization rates in refugees, indicating unexplained physical symptoms occurring frequently, also due to mental health stigma [9, 12, 13].

### Predicting factors for mental health problems in refugees

The factors predicting refugees‘ mental health are often long-term or have an impact on different phases of migration. Therefore, categorization is a challenge [14]. In order to give an overview, we defined the categories I) sociodemographic and flight-independent factors, II) war- and flight-related factors and III) post-migration factors. Since few studies examine the specific target group of Syrian refugees, most of the studies reported below focused on refugees from and settled in different countries.

#### Sociodemographic and flight-independent factors

Female sex and older age are well documented predicting factors for mental distress in refugees, including positive associations with PTSD, depression, anxiety and low subjective well-being [10, 11, 15–20]. In contrast, Tekeli-Yesil et al. [21] found younger age to be associated with higher frequencies of PTSD. Regarding family status, being single, divorced, widowed or living apart from one’s partner were found to be risk factors, while being married was found to be a protective factor for PTSD, which could highlight the importance of social support [10, 14]. Higher pre-displacement socioeconomic status was shown to be a predictor of mental distress in refugees [20]. A lower post-migration economic status in males was a predictor for depression [17]. While higher education was associated with worse mental health outcomes in a study by Porter and Haslam, [20] low education was stated as a predicting factor for mood and anxiety disorders by Bogic et al. [22]. Tinghög et al. [10] found no association between education and distress. Differences between refugee populations and post-migration conditions are a possible explanation for the diverse findings. Also, one study showed that internalized mental health stigma seems to be a risk-factor for developing PTSD [23].

#### War- and flight-related factors

The experience of a larger variability of TE [Note: We use the term “variability of TE” instead of the common expression “sum/number of TE” since it is not the number of experiences that is assessed but the different types of traumatization.] is a predicting factor for PTSD, depression and anxiety in refugees [11, 17, 18], with a special focus on interpersonal violence [14, 15]. TE can occur in all phases of migration, during the war in Syria, on flight-routes (e.g., unsafe means of transport) as well as in the country of arrival (e.g., detention) [13]. Georgiadou et al. [11] found a shorter duration of the flight associated with higher depression, in contrast to Nesterko et al. [16], who did not find any associations between flight-related factors and symptom burden. Tekeli-Yesil et al. (2018) reported flight to another country per se to predict higher depression [21].

#### Post-migration factors

It is widely discussed that post-migration factors have a major impact on refugees’ mental health [10, 24–26]. In addition to the assumption that post-migration factors may represent more important risk or protective factors than sociodemographic or pre-migratory factors, they could also moderate the ability of recovery from pre-migration trauma [21, 27]. Syrian refugees in Germany named language-skills, socioeconomic living conditions, concern for and being in contact with family, discrimination and unclear asylum procedures as important factors for psychological adaption [28]. Tinghög et al. [10] found economic strain, perceived discrimination, feelings of isolation, missing social life in home country, participation barriers (e.g., communication problems) and family conflicts to be predictors of mental distress in Syrian refugees resettled in Sweden. Concerns about home country and family were shown to be a predicting factor for mental distress in refugees [10, 14, 20, 25]. In contrast to a study by Schweitzer et al., [25] lack of social support was not found as a predicting factor in this study, even though Syrian refugees in Germany named social support as an important coping mechanism [29]. In addition, Colic-Peisker and Walker [30] highlighted the importance of the loss of social identity (e.g., roles, status) for refugees arriving in a new country.

Due to huge differences in countries of arrival (e.g., resettlement policies, socioeconomic inclusiveness, supply structure), it is necessary to specifically analyze post-migration factors in different settings [14, 22]. According to our knowledge, there is a lack of studies focusing on Syrians as the largest group of refugees in Europe’s main country of arrival. In this regard, Syrian refugees with posttraumatic stress symptoms (PTSS) constitute a vulnerable subgroup for whom a more detailed examination of predictors on mental health may yield important clinical implications. Hence, the aim of this study is to investigate the contribution of the above-mentioned predictors on mental health in Syrian refugees with PTSS based in Germany.

## Methods

### Study sample and data collection

We used baseline data of the randomized controlled trial “Help@App: Development and evaluation of a self-help app for traumatized Syrian refugees in Germany” (i.e., the SANADAK trial). The project was approved by the Ethics committee of the Medical Faculty of the University of Leipzig, Germany (ID: 111–17-ek). Data was collected between 11/2018 and 12/2019, focusing on Syrian refugees living in the urban area of Leipzig, Halle/Saale and Dresden, who were recruited using a multi-strategic approach. Inclusion criteria for the trial were: Syrian refugee aged between 18–65 years with the experience of at least one TE and mild to moderate PTSS (PDS-5 = 11–59) as well as owning a device compatible of running the self-help app applied in the project “Help@App”. Exclusion criteria were: Severe posttraumatic stress symptomatology (PDS-5 ≥ 60), severe depression (PHQ-9 ≥ 20), acute suicidality (Depressive Symptom Inventory-Suicidality Subscale/DSI-SS ≥ 3; [31]), current psychotherapy, psychiatric treatment, psychopharmaceutic medication and pregnancy. There were no differences between the recruitment and the trial sample regarding almost all clinical and sociodemographic characteristics [32]. Data was collected using structured interviews, conducted by trained Arabic native speakers. Further information on the project and the sample selection can be found elsewhere [33, 34].

### Instruments

A questionnaire including sociodemographic and flight-related questions and standardized instruments covering symptoms of PTSD, depression, somatization and anxiety was used. The questionnaire was translated based on the TRAPD model (Translation, Review, Adjudication, Pretesting, and Documentation; [35]) and pretested by a group of bilingual experts.

#### Sociodemographic and flight-related questions

Participants were asked about their age, sex, family status, education, living conditions, residential status and occupational status. Concerning the flight-process, participants were asked about the loss or going missing of a close person, the duration of the flight, the means of escape and the connection to their home country.

#### Main measures and predictors

Anxiety was assessed with the GAD-7 (Generalized Anxiety Disorder Screener; Spitzer et al., 2006 [36]). The instrument has 7 items and uses a 4-point Likert scale from 0 (not at all) to 3 (nearly every day) per item to measure anxiety symptoms such as “feeling of fear, as if something bad is going to happen”. The maximum score is 21 points with a higher score indicating higher symptom burden. A cutoff of ≥10 marks a probable anxiety diagnosis (Kroenke et al., 2010 [37]). Internal consistency (Cronbach’s α) in the present study was .846.

The PHQ-9 (Patient Health Questionnaire 9; Kroenke et al. 2001 [38]) was applied to measure depressive symptoms. It consists of 9 items with a 4-point Likert scale from 0 (not at all) to 3 (nearly every day), inquiring about symptoms such as "dejection, melancholy, or hopelessness". Maximum is 27 points, with a higher score indicating higher symptom burden. A cutoff of ≥10 indicate a probable depression diagnosis (Kroenke et al., 2010 [37]). Internal consistency (Cronbach’s α) in the present study was .809.

Posttraumatic Stress Disorder was assessed using the PDS-5 (Posttraumatic Diagnostic Scale-5; Foa et al., 1997 [39]). The 24-item-instrument measures posttraumatic stress symptoms such as “unwanted, stressful memories of the trauma” on a 5-point Likert scale from 0 (not at all) to 4 (6 or more times a week/severe). The maximum score is 80, with a higher score indicating a higher symptom burden. A probable PTSD diagnosis is marked over a cutoff of ≥28 (Foa et al., 2016 [40]). Internal consistency (Cronbach’s α) in the present study was .838. Additionally, the traumatic events assessed with the PDS-5 provided the possibility to examine the total variability of TE.

The PHQ-15 (Patient Health Questionnaire-15; Kroenke et al., 2002 [41]) was used to measure symptoms of somatization. The instrument originally consists of 15 items and a 3-point Likert scale from 0 (not at all) to 2 (more than half the days or nearly every day). For example, the items inquire “headache” or “chest pain”. The item “menstrual cramps or problems with your periods” was removed to avoid gender bias in the outcome, leading to a total score of 28 instead of the original 30 [42]. Higher scores represent higher symptom burden and a cutoff of ≥10 indicates a probable diagnosis of somatization (Kroenke et al., 2010 [37]). Internal consistency (Cronbach’s α) in the present study was .821.

The GSE (Generalized Self-Efficacy Scale; Schwarzer & Jerusalem, 1995 [43]) was implemented to measure a person’s expectation of being able to deal competently with challenging situations. The instrument consists of 10 items measured on a 4-point Likert scale ranging from 1 (not true) to 4 (true exactly). An example item reads “Even in the case of surprising events, I believe I can cope with them well”. The total score is 40 points, with higher scores indicating higher generalized self-efficacy. Internal consistency (Cronbach’s α) in the present study was .821.

Religiousness was assessed using the Z-Scale (Huber, 2003 [44]). The 10-item-scale consists of a 5-point Likert scale ranging from 0 (not at all) to 4 (very) with items like “How religious would you describe yourself as being?”. The total score is 20, with higher scores indicating stronger religiousness. Internal consistency (Cronbach’s α) in the present study was .854.

Social support was measured using the ESSI (ENRICHD Social Support Inventory; Kendel et al., 2011 [45]. The 5-point Likert scale consists of 5 items ranging from 1 (never) to 5 (always). An example item reads “Is there someone available to you who can give you good advice when you have problems?”. The total score is 25, with a higher score indicating the perception of more social support. Internal consistency (Cronbach’s α) in the present study was .820.

Mental health stigma was assessed using the SSMIS-SF (Self-Stigma of Mental Illness Scale–Short Form; Corrigan et al., 2012 [46]). The 4 dimensions of mental health stigma that are measured are “stereotype awareness”, “agreement”, “apply to self” and “harm to self”. The scale consists of 20 items (5 in each dimension) using a 10-point Likert scale ranging from 1 (I do not agree at all) to 9 (I completely agree). An example item from the dimension “stereotype awareness” reads “From my perspective, the public believes most people with mental illness are dangerous”. The total is calculated individually for each dimension and can reach a maximum of 45 each, with higher scores indicating higher mental health stigma. Internal consistency (Cronbach’s α) in the present study was .659 for dimension “stereotype awareness”, .574 for dimension “agreement”, .550 for dimension “apply to self” and .737 for dimension “harm to self”.

Overall life-satisfaction was measured in one item on an 11-point scale ranging from 0–10 (*Highly unsatisfied—Highly satisfied*). Furthermore, we assessed the connection to the country of origin with a 5-point scale ranging from 1–5 (*Very strong—Not at all*).

### Statistical analysis

Statistical analyses were conducted using IBM-SPSS 25.0 statistical package for Windows (IBM, 2017). Descriptive analyses were carried out to give an overview of the sample characteristics and mental health burden in the main outcomes PDS-5, PHQ-9, PHQ-15 and GAD-7. Multiple linear regression analyses were conducted to examine predictors using the enter method. In the first step, sociodemographic variables were included, followed by potential risk and protective factors (i.e., connection to country of origin, religiousness, social support, variability of TE, self-efficacy, stigma, life satisfaction) in a second step. We chose a hierarchical procedure for controlling sociodemographic variables in a first step, while adding potential predictors in a second step. After checking for regression assumptions (linearity, multivariate normality, multicollinearity, auto-correlation, homoscedasticity, normal distribution of residuals), we found a violation of homoscedasticity and thus used bootstrapping with 2000 iterations for calculating 95% confidence intervals (95% CIs) for the outcomes of GAD-7, PDS-5, and PHQ-15. For replicability of calculations, SPSS MT (Mersenne Twister) was applied.

## Results

### Sample characteristics

The recruitment sample consisted of 170 adult Syrian refugees settled in Germany. The participants were reached through contacts with facilities highly frequented by Syrian refugees (e.g., psychosocial centers, language schools, community colleges). After a screening for eligibility, 37 persons (21.8%) were excluded and thus a total of 133 participants (78.2%) participated in the present study. Details on the inclusion process and drop-outs can be found in Röhr et al. [32].

### Sociodemographic and migration-related data

Table 1 gives an overview of sociodemographic data while Table 2 shows details of flight-related sample characteristics. The mean age of participants was 33.34 years (*SD* = 11.18). Most participants were male (n = 82; 61.7%) and had an average education of 13.82 (*SD* = 3.96) years including vocational training or university studies. More than half of the participants were single (n = 69; 51.9%) and had spent an average time of 42.16 (SD = 13.95) months in Germany.

**Table 1:** Sociodemographic variables.

Characteristics	n (%)
Age	
M/*SD*/range	33.34/11.18/18-64
Sex	
Male	82 (61.7)
Female	51 (38.3)
School education (in years)	
M/*SD*/range	13.82/3.96/0-24
Family status	
Single	69 (51.9)
Married	51 (38.3)
Divorced	7 (5.3)
Widowed	3 (2.3)
I don’t know	3 (2.3)
Housing situation	
Alone	33 (24.8)
With others	100 (75.2)
Residential status	
Tolerance of stay (Duldung)	0 (0.0)
In asylum procedure (Gestattung)	15 (11.3)
Residency permit (Erlaubnis)	108 (81.2)
Other	8 (6.0)
Employment	
Unemployed	89 (67.9)
Employed	42 (21.1)
Income	
< = 500€	26 (19.5)
> 500€	107 (80.5)

*Note*. N = 133 adult Syrian refugees in Germany.

**Table 2:** Flight-related variables.

Characteristics	n (%)
Connection to country of origin	
Very Strong	41 (30.8)
Strong	33 (24.8)
Partly	28 (21.1)
Little	22 (16.5)
Not at all	9 (6.8)
Missing of a close person	
Yes	47 (35.3)
No	86 (64.7)
Additional loss of a close person*	
Yes	61 (45.9)
No	4 (3.0)
No answer	1 (0.8)
Duration of flight (in months)	
M/*SD*/range	9.20/15.32/0-66
Means of escape (multiple answers possible)	
By airplane	58 (43.6)
Overland route	86 (64.7)
Oversea route	76 (57.1)
Via transit country**	23 (17.3)
By other means	1 (0.8)

*Note*. N = 133 adult Syrian refugees in Germany

*n = 66 (only those missing a close person were asked)

**duration of stay ≥ 3 months.

### Predictors, overall symptom severity, variability of TE and comorbidities

Descriptive statistics for predictors can be seen in S1 Table, detailed outcome categories for the measures PHQ-9, PHQ-15 and GAD-7 can be seen in S2 Table. The total score for PTSD was M = 23.82 (*SD* = 11.61), with 30.8% (n = 41) ≥ 28 points. For depression, the total score was M = 9.25 (*SD* = 5.24), with 45.1% (n = 60) ≥ 10 points. For generalized anxiety, the total score was M = 8.54 (*SD* = 4.97), with 40.6% (n = 54) ≥ 10 points. For somatization, the total score was M = 8.69 (*SD* = 5.19) with 46.6% (n = 62) ≥ 10 points. Concerning comorbidities, 34.6% (n = 46) of the participants did not score above any cutoff, 19.5% (n = 26) scored above the cutoff in one measure, 14.3% (n = 19) in two, 11.3% (n = 15) in three and 20.3% (n = 27) in four measures. In total, 65.4% (n = 87) scored above the cutoff in at least one measure. Correlations between the measures PDS-5, PHQ-9, PHQ-15 and GAD-7 and a detailed overview of the comorbidities are given in S2 and S3 Tables. Participants experienced M = 3.11 TE (*SD* = 1.62). The most frequently mentioned TE were "combat mission in war or have lived in a war zone" with 83.49% (n = 111), “physical violence” with 50.37% (n = 67) and “accidents” with 35.33% (n = 47). More information on TE and a split by gender can be found in S5 Table.

### Regression models

Results of the bootstrapped regression models for PTSD, somatization and anxiety as well as the regression model for depression are presented in Table 3.

**Table 3:** Results from multiple linear hierarchical regression analysis (Model 2).

	PTSD	Somatization	Anxiety	Depression
	*B* [*SE*; 95% BCaCI] ^†^	*B* [*SE*; 95% BCaCI] ^†^	*B* [*SE*; 95% BCaCI] ^†^	*B* [*SE B;* β]
Step 1: Sociodemographic variables				
Age	-0.01 [0.10; -0.20, 0.20]	0.06 [0.05; -0.03, 0.16]	-0.01 [0.05; -0.10, 0.09]	0.00 [0.04; 0.01]
Sex (1 = male; 2 = female)	3.20 [2.30; -1.26, 7.61]	2.34 [1.11; 0.30, 4.25]*	0.88 [1.08; -1.14, 2.77]	2.33 [0.98; 0.22]*
School Education (in years)	0.15 [0.26; -0.38, 0.67]	-0.16 [0.13;-0.43, 0.13]	0.00 [0.11; -0.21, 0.23]	0.22 [0.11; 0.17]*
Living alone	-2.29 [2.45; -7.02, 1.99]	-0.30 [1.02; -2.19, 1.38]	-0.19 [1.05; -2.30, 1.60]	-0.36 [0.99; -0.03]
Employment	-1.37 [2.10; -5.40, 2.93]	-0.91 [1.05; -2.91, 1.15]	-1.70 [0.95; -3.59, 0.17]	-1.51 [0.99; -0.14]
Income < 500€ (0 = no; 1 = yes)	7.04 [2.90; 0.79, 13.72]*	1.11 [1.12; -1.21. 3.68]	0.88 [1.20; -1.34, 3.54]	0.64 [1.07; 0.05]
ΔR^2^ step 1	.173**	.146**	.097	.126*
Step 2: Risk and protective factors				
Connection to country of origin	-0.71 [0.82; -2.24, 0.81]	0.24 [0.34; -0.44, 0.88]	-0.59 [0.32; -1.25, 0.02]	-0.80 [0.33; -0.20]*
Religiousness	0.12 [0.16; -2.14, 0.47]	-0.21 [0.08; -0.19, 0.14]	0.07 [0.07; -0.08, 0.22]	-0.03 [0.07; -0.03]
Social Support	-0.55 [0.23; -0.98, -0.05]*	-0.11 [0.11; -0.31, 0.12]	-0.20 [0.09; -0.39, 0.01]*	-0.35 [0.10; -0.32]***
Variability of traumatic events	1.15 [0.58; -0.08, 2.56]	0.95 [0.26; 0.41, 1.55]**	0.13 [0.26; -0.42, 0.73]	0.52 [0.25; 0.17]*
Self-efficacy	-0.25 [0.25; -0.81, 2.66]	-0.06 [0.10; -0.26, 0.11]	-0.02 [0.11; -0.23, 0.16]	0.01 [0.09; 0.01]
Stigma: Agreement	0.39 [0.16; 0.05, 0.70]*	0.15 [0.10; -0.06, 0.34]	0.05 [0.08; -0.13, 0.21]	0.07 [0.07; 0.09]
Stigma: Apply to self	-0.05 [0.18; -0.42, 0.36]	-0.03 [0.08; -0.19,0.16]	0.16 [0.07; 0.00, 0.33]*	0.18 [0.07; 0.23]*
Life Satisfaction	-0.45 [0.51; -1.48, 0.57]	-0.21 [0.23; -0.65, 0.23]	-0.14 [0.22; -0.55, 0.31]	-0.48 [0.20; -0.21]*
ΔR^2^ step 2	.155**	.124*	.122*	.243***
Total R^2^ (Adjusted R^2^)	.328 (.243)**	.270 (.178)*	.219 (.120)*	.369 (.289)***

*Note*. PTSD (PDS-5), Somatization (PHQ-15), Anxiety (GAD-7), Depression (PHQ-9); *B* = unstandardized coefficient; *SE* = Standard Error; BCaCI = Bias-corrected and accelerated 95% Confidence Interval; R^2^ (R square) = percentage of variance explained by the model; AdjR^2^ (adjusted R square) = adjusted for the number of terms included; ΔR^2^(Delta R Square) = Change in R square; *SE B* = coefficients standard error; β = standardized coefficient

^†^ = bootstrapped regression

*p < 0.05.

**p < 0.01.

***p < 0.001; n = 126.

#### PTSD

Income less than € 500 /month (*B* = 7.04, *p* < .05), less social support (*B* = -0.55, *p* < .05) and agreement with mental illness stereotypes (*B* = 0.39, *p* < .05) were statistically significantly associated with higher severity of PTSD. These variables accounted for 32.8% of the variance (R^2^ = .328).

#### Depression

Statistically significant predictors for a higher severity of depression were female sex (β = .22, *p* < .05), higher number of school years (β = .17, *p* < .05), higher connection to home country (β = -.20, *p* < .05), less social support (β = -.32, *p* < .001), higher variability of TE (β = .17, *p* < .05), applying mental health stigma to oneself (β = .23, *p* < .05), and less life satisfaction (β = -.21, *p* < .05). Overall, these variables accounted for 36.9% of the variance (R^2^ = .369).

#### Somatization

Female sex (*B* = 2.34, *p* < .05) and a higher variability of TE (*B* = 0.95, *p* < .01) statistically significantly predicted higher severity of somatization, accounting for 27.0% of the variance (R^2^ = .270).

#### Anxiety

Less social support (*B* = -0.20, *p* < .05) and applying stigma to oneself (*B* = 0.16, *p* < .05) were statistically significantly associated with higher severity of anxiety. Overall, these variables accounted for 21.9% of the variance (R^2^ = .219).

## Discussion

The aim of this study was to investigate sociodemographic, war- and flight-related, and post-migration factors as predictors of posttraumatic stress, depression, somatization and anxiety in Syrian refugees with PTSS based in Germany.

Although our sample consisted of help-seeking individuals with PTSS, the prevalence rates of mental distress (PTSD, depression, anxiety) are comparable to the population-based survey of Syrian refugees in Sweden by Tinghög et al. [10] On this basis, we argue that even though our sample is not representative, the high prevalence of somatization (46.6%) highlights the importance of this disorder as manifestation of mental distress in Syrian refugees. This should be addressed in further research.

Our findings suggest that sociodemographic (i.e., female sex, higher education) and flight-related predicting factors (i.e., variability of TE) have a negative impact on mental health in Syrian refugees with PTSS in Germany. An important finding is that mental health stigma predicts distress in almost all investigated mental health outcomes. Post-migration factors have a major impact on mental health, such as low income, lack of social support, low life satisfaction and a strongly felt connection to Syria.

### Sociodemographic and flight-unrelated factors

Female sex, higher education and mental health stigma showed to be predictors for mental distress in Syrian refugees with PTSS. In line with other research, our study found female sex to be a predicting factor for somatization and depression [10, 14, 17]. Female sex was no predicting factor for PTSD and anxiety. Regarding PTSD, this is in line with previous research in the refugee population in Germany [11, 14]. However, in contrast, these same previous studies found female sex to predict anxiety. The difference to the result from Nesterko et al. [14] could be attributed to the broader target group and the use of different instruments. However, this is not the case in the study by Georgiadou et al. [11], which also used the GAD-7 in a group of Syrian refugees in Germany. Hence, further research is needed on the extent to which female sex predicts anxiety. As stated in previous research [47], our findings support the hypothesis that flight and resettlement are gendered experiences with women* [Note: The gender star indicates the constructed character of "gender". It means that the term "women" refers to all persons who define themselves as such] facing special stress factors, which should be taken into account, both in support services and politically.

We found higher education as a predicting factor for depression. Porter and Haslam [20] reported this association earlier and hypothesized that it might be related to a greater loss of status in the country of arrival. As Colic-Peisker and Walker [30] argued similarly, the role of social identity and loss of status should be subject to further research.

Interestingly, aspects of mental health stigma (i.e., agreement to stigma-beliefs in society, apply of societal stigma-beliefs to self) seem to be predicting factors for PTSD, depression and anxiety. So far, the main research in this field focused on mental health stigma as a barrier to seeking help [29, 48, 49], but rarely on stigma as predicting factor for mental distress. Hence, our results strengthen the scarce evidence in previous literature regarding stigma as an important predicting factor for mental distress in refugees with PTSS. These findings are especially important, since mental health stigma scores tend to be high in refugees [23].

Age and family status were not found as predicting factors for any outcome in our study, which is in contrast to previous literature [10, 11, 16, 21, 50] and could be due to our sample mainly consisting of relatively young participants, leading to a lack of variability in the higher age range. Regarding family status, being single as predicting factor might be levelled out to a certain degree by the living situation, since most of our participants lived in multi-person households.

### War- and flight-related factors

Concerning flight-related factors, we found a higher variability of TE to predict higher depression, which is in line with Tinghög et al. [10] and Kaya et al. [17]. Nevertheless, higher variability of TE did not predict higher PTSD or anxiety in our study, contrasting various studies postulating predictive value for PTSD and anxiety [10, 17, 18]. This could be explained by a lack of variability in our sample, as all participants had experienced at least one TE and had a PDS-5 score of at least 11, based on our inclusion criteria. Analogous to Nesterko et al., [14] we found no correlation between flight-specific factors (e.g., duration of escape, means of transport) and mental distress. Taking into account the extent of traumatization caused by civil war, it is possible that flight-specific factors may no longer play a major role in the development or severity of mental distress, since the onset could have already occurred earlier.

### Post-migration factors

We identified a number of post-migration factors as predictors for the mental health of Syrian refugees’ mental. An important finding was that an income <€ 500 /month predicted higher PTSD, which is in contrast to previous studies [21, 51]. To put the value in relation, the at-risk-of-poverty threshold for Germany in 2016 was € 1064 /month for an adult living alone [52]. No statistically significant effect of income was shown regarding depression, also contradicting the study by Nicholson [51]. Different cutoffs, times and settings of the studies might be responsible for the diverse findings. Our results are supported by studies that investigated the risk of PTSD following terrorism or natural disasters reporting low income as predicting factor for PTSD in non-refugees after TE [53, 54].

A lack of social support was associated with higher PTSD, depression and anxiety. Our findings are in line with our previous research, where social support was named as most important coping strategy by Syrian refugees in Germany [29]. Nevertheless, there are ambiguous findings in the literature regarding the role of social support [10, 25], and further research should explore which forms of social support are helpful.

Another predicting factor for depression was a low overall life satisfaction in the post-migration situation. This finding seems intuitive and supports previous research [54–56]. We further found a strongly-felt connection to Syria as a predicting factor for depression. Following the Conservation of Resources Model by Hobfoll et al., [57] this could be attributed to a higher vulnerability after TE due to multiple losses connected with leaving Syria.

### Loss

On closer inspection, many of the predicting factors we identified can be seen in connection with loss: Loss of status, especially for people with higher education, loss of income and loss of home including the social network. For example, this was reflected in our sample in the observation that the majority of participants were highly educated while 67.9% were unemployed. These findings add to studies focusing on interpersonal losses [58–60] and highlight the necessity to treat loss as an important issue for refugees’ mental health and to derive practical implications.

### Clinical implications

Post-migration factors represent important predicting factors for the mental health of Syrian refugees with PTSS and are modifiable on a policy level by Germany as country of arrival, such as securing financial security and promoting gender equality–which also means giving special support to women* refugees as a vulnerable group (e.g., psychoeducation about somatization in groups of women*). Tailored psychosocial programs for refugees with PTSS should address mental health stigma and build social support networks. The issue of loss should play an important role in non-pathologizing, resource-oriented support programs, validating the extreme experiences that refugees from Syria have made during war and flight. These measures not only signify steps towards the observance of human rights, they also lead to a relief of the medical infrastructure and to a better chance for the new arrivals to contribute their resources to society.

### Strengths and limitations

A strength of this study is the very specific target group of Syrian refugees. Many studies in the field include people from very diverse political and cultural contexts, only united by the concept of the “refugee”, possibly leading to unwanted homogenization. Though our sample is not representative, its demographic characteristics are similar to the general Syrian refugee population in Germany regarding gender and age [61] and can hence give important insights into the predicting factors for the biggest subgroup of refugees in Germany. Furthermore, we included post-migration factors such as living conditions in the analysis, in an attempt to depict the current life reality of those affected.

A limiting factor is that as it was necessary for the “SANADAK trial”, only Syrian refugees with at least mild PTSS and no severe PTSD or depression were included in our study. Although the very specific sample leads to a high predictive value of the results, it severely limits their generalizability. The reduced variability in the range of mental health burden could have led to a possible underestimation of the effect of predictors in our study. Other limitations and possible sources of bias could be the cross-sectionality of our analyses and the use of self-reported data in our study. Generalization of results is additionally limited due to strong impact of post-migration factors that may be specific for Germany or at least European high-income countries. Furthermore, sex was only recorded in binary form and gender was not assessed at all, meaning that experiences of some particularly vulnerable groups (e.g., LGBTQIA+) could not be depicted.

Future research should try to depict the reality of different people’s lives in terms of gender as comprehensively as possible. More research on somatization as a manifestation of mental distress in refugees would also be desirable. A broader range of post-migration factors such as language barriers, experiences of racism, substance abuse and physical illness should also be examined. Furthermore, a more detailed research on the topic of loss (including an assessment of loss in instruments addressing TE) and an assessment not only of the variability of TE but also the actual number of experienced TE could contribute to a better understanding of the refugee experience.

## Conclusion

Our study showed a range of predicting factors for the mental health of Syrian refugees with PTSS. Sociodemographic predictors such as female sex and higher education as well as flight-related predictors such as a higher variability of TE had a negative impact on mental health in Syrian refugees with PTSS in Germany. Mental health stigma was shown to be a predicting factor for PTSD, depression and anxiety. Post-migration factors had a major impact on mental health, such as low income, lack of social support, low life satisfaction or a strongly-felt connection to Syria. Our findings indicate that somatization is an important manifestation of mental distress in Syrian refugees with PTSS, predicted by female sex and a higher variability of TE. Further research should widen the perspective to the topics of loss, gender diversity and post-migration factors such as participation barriers or racism.

## Supporting information

S1 Table Predictors and measures of mental distress. (DOCX)

S2 Table Symptom severity. (DOCX)

S3 Table Pearson correlations of the main mental health outcomes. (DOCX)

S4 Table Prevalence of mental distress. (DOCX)

S5 Table Traumatic events by gender. (DOCX)
