# Supplementary material for: Predictors of psychological distress in Syrian refugees with posttraumatic stress in Germany
Source: PLoS One. 2021 Aug 4;16(8):e0254406. doi: 10.1371/journal.pone.0254406 (PMC8336813; doi:10.1371/journal.pone.0254406)
Supplement: S1 Table — (DOCX) [file pone.0254406.s001.docx]

**S1 Table.** Predictors and measures of mental distress.

| Characteristics | n | M/*SD*/Range |
| --- | --- | --- |
| Religiousness (Z-Scale) | 130 | 11.60/6.22/0-23 |
| Social support (ESSI) | 133 | 18.01/4.71/5-25 |
| Variability of traumatic events (PDS-5 event scale) | 133 | 3.11/1.62/1-7 |
| Self-efficacy (GSE) | 132 | 27.38/4.67/14-38 |
| Stigma: Agreement (SSMIS Dim. II) | 131 | 18.82/6.73/5-38 |
| Stigma: Apply to self (SSMIS Dim. III) | 132 | 16.52/6.60/5-43 |
| Life Satisfaction (PGI) | 130 | 23.74/6.56/9-37 |
| PTSD (PDS-5) | 133 | 23.82/11.61/6-59 |
| Depression (PHQ-9) | 133 | 9.25/5.24/0-24 |
| Somatization (PHQ-15) | 133 | 8.69/5.19/0-24 |
| Anxiety (GAD-7) | 133 | 8.54/4.97/0-21 |

*Note.* N = 133 adult Syrian refugees in Germany; *M* = mean value; *SD* = standard deviation; Range = total
range (minimum - maximum).
