# Supplementary material for: Predictors of psychological distress in Syrian refugees with posttraumatic stress in Germany
Source: PLoS One. 2021 Aug 4;16(8):e0254406. doi: 10.1371/journal.pone.0254406 (PMC8336813; doi:10.1371/journal.pone.0254406)
Supplement: S2 Table — (DOCX) [file pone.0254406.s002.docx]

**S2 Table.** Symptom severity.

| Measures | n | % |
| --- | --- | --- |
| Depression (PHQ-9) |  |  |
| None-Minimal (0-4) | 29 | 21.8 |
| Mild (5-9) | 44 | 33.1 |
| Moderate (10-14) | 32 | 24.1 |
| Moderately severe (15-19) | 26 | 19.5 |
| Severe (20-27) | 2 | 1.5 |
| Somatization (PHQ-15) |  |  |
| None-Minimal (0-4) | 32 | 24.1 |
| Mild (5-9) | 39 | 29.3 |
| Moderate (10-14) | 46 | 34.6 |
| Severe (15-27) | 16 | 12.0 |
| Generalized Anxiety (GAD-7) |  |  |
| None-Minimal (0-4) | 34 | 25.6 |
| Mild (5-9) | 45 | 33.8 |
| Moderate (10-14) | 35 | 26.3 |
| Severe (15-21) | 19 | 14.3 |

*Note.* N = 133 adult Syrian refugees in Germany.
