# Supplementary material for: Predictors of psychological distress in Syrian refugees with posttraumatic stress in Germany
Source: PLoS One. 2021 Aug 4;16(8):e0254406. doi: 10.1371/journal.pone.0254406 (PMC8336813; doi:10.1371/journal.pone.0254406)
Supplement: S3 Table — (DOCX) [file pone.0254406.s003.docx]

**S3 Table.** Pearson correlations of the main mental health outcomes.

|  | PTSD (PDS-5) | Depression (PHQ-9) | Somatization (PHQ-15) | Anxiety (GAD-7) |
| --- | --- | --- | --- | --- |
| Depression (PHQ-9) | .675* | - |  |  |
| Somatization (PHQ-15) | .547* | .582* | - |  |
| Anxiety (GAD-7) | .619* | .822* | .586* | - |

*Note:* N = 133 adult Syrian refugees in Germany; **p* < 0.001.
