# Supplementary material for: Predictors of psychological distress in Syrian refugees with posttraumatic stress in Germany
Source: PLoS One. 2021 Aug 4;16(8):e0254406. doi: 10.1371/journal.pone.0254406 (PMC8336813; doi:10.1371/journal.pone.0254406)
Supplement: S4 Table — (DOCX) [file pone.0254406.s004.docx]

**S4 Table.** Prevalence of mental distress.

| Number of Diagnoses |  | n (%) |
| --- | --- | --- |
| Single diagnostic category |  |  |
| Only PTSD |  | 4 (3.0) |
| Only depression |  | 6 (4.5) |
| Only anxiety |  | 3 (2.3) |
| Only somatization |  | 13 (9.8) |
| Two diagnostic categories |  |  |
| PTSD and depression |  | 2 (1.5) |
| PTSD and anxiety |  | 0 (0.0) |
| PTSD and somatization |  | 1 (0.8) |
| Depression and anxiety |  | 7 (5.3) |
| Depression and somatization |  | 6 (4.5) |
| Somatization and anxiety |  | 3 (2.3) |
| Three diagnostic categories |  |  |
| PTSD and somatization and depression |  | 1 (0.8) |
| PTSD and anxiety and depression |  | 3 (2.3) |
| PTSD and anxiety and somatization | | 3 (2.3) |
| Depression and somatization and anxiety |  | 8 (6.0) |
| Four diagnostic categories |  |  |
| Depression and somatization and anxiety and PTSD |  | 27 (20.3) |

*Note.* N = 133 adult Syrian refugees in Germany; PTSD (PDS-5) ≥28; depression (PHQ-9) ≥ 10;
somatization (PHQ-15) ≥ 10; anxiety (GAD-7) ≥ 10.
