# Supplementary material for: Predictors of psychological distress in Syrian refugees with posttraumatic stress in Germany
Source: PLoS One. 2021 Aug 4;16(8):e0254406. doi: 10.1371/journal.pone.0254406 (PMC8336813; doi:10.1371/journal.pone.0254406)
Supplement: S5 Table — (DOCX) [file pone.0254406.s005.docx]

**S5 Table.** Traumatic events by gender.

| **Traumatic event** | **Male**  n (%) | **Female**  n (%) | **Total**  n (%) |
| --- | --- | --- | --- |
| Severe, life-threatening disease | 21 (25.61) | 14 (27.45) | 35 (26.32) |
| Physical violence (e.g. assaulted with a weapon, serious injury from fighting, threatened with firearm, etc.) | 50 (60.98) | 17 (33.33) | 67 (50.38) |
| Sexualized violence (e.g. (attempted) rape, forced sexual intercourse under threat of a weapon, etc.) | 3 (3.66) | 4 (7.84) | 7 (5.26) |
| Combat mission in war or have lived in a war zone | 68 (82.93) | 43 (84.31) | 111 (83.49) |
| Child abuse (severe beatings, sexual acts with a person at least 5 years older, etc.) | 19 (23.17) | 9 (17.65) | 28 (21.05) |
| Accident (serious injury or death due to car accident, work accident, house fire, etc.) | 33 (40.24) | 14 (27.45) | 47 (35.33) |
| Torture or imprisonment | 29 (35.37) | 9 (17.65) | 38 (28.57) |
| Natural disaster (hurricane, flood, earthquake, etc.) | 1 (1.22) | 0 (0) | 1 (0.75) |
| Other (e.g. death of relatives, sight of war corpses, attempted kidnapping, etc.). | 52 (63.41) | 27 (52.94) | 79 (59.40) |

*Note.* N = 133 adult Syrian refugees in Germany; male participants n = 82, female participants n = 51; traumatic events
based on the PDS-5 (Foa et al., 1997).
